# Supplementary material for: Primary health care interventions targeting diabetes, hypertension or dyslipidemia in Malaysia: A scoping review
Source: PLoS One. 2026 Apr 20;21(4):e0346934. doi: 10.1371/journal.pone.0346934 (PMC13095119; doi:10.1371/journal.pone.0346934)
Supplement: S2 Table — (DOCX) [file pone.0346934.s002.docx]

## **S2 Table. Search strategies**

1. **MEDLINE**

| **Terms** | **Results** |
| --- | --- |
| ("diabetes mellitus, type 2"[MeSH Terms] OR "diabet*"[Title/Abstract] OR "type 2 diabetes"[Title/Abstract] OR "type II diabetes"[Title/Abstract] OR "hyperglycemia"[Title/Abstract] OR "hyperglycaemia"[Title/Abstract] OR "high blood glucose"[Title/Abstract] OR "NIDDM"[Title/Abstract] OR "noninsulin dependent diabetes mellitus"[Title/Abstract] OR "non insulin dependent diabetes mellitus"[Title/Abstract] OR "glucose metabolism disorders"[Title/Abstract] OR "dysglycemia"[Title/Abstract] OR "dysglycaemia"[Title/Abstract] OR "impaired glucose tolerance"[Title/Abstract] OR "impaired fasting glucose"[Title/Abstract] OR "maturity onset diabetes of the young"[Title/Abstract] OR "MODY"[Title/Abstract] OR "T2DM" OR "maturity onset diabetes"[Title/Abstract] OR "impaired fasting blood sugar"[Title/Abstract]) **OR** ((((hypertension[Title/Abstract]) OR ("high blood pressure"[Title/Abstract])) OR ("raised blood pressure"[Title/Abstract])) OR (hyperten*[Title/Abstract])) OR ("Hypertension"[Mesh]) **OR** ("dyslipid*"[Title/Abstract] OR "hypercholesterol*"[Title/Abstract] OR "hyperlipid*"[Title/Abstract] OR "Hypercholesterolemia"[MeSH Terms] OR "Dyslipidemias"[MeSH Terms]) **AND** "community health centers"[MeSH Terms] OR "community medicine"[MeSH Terms] OR "ambulatory care facilities"[MeSH Terms] OR "ambulatory care"[MeSH Terms] OR "mobile health units"[MeSH Terms] OR "primary health care"[MeSH Terms] OR "primary health care"[Title/Abstract] OR "primary care"[Title/Abstract] OR "public health"[Title/Abstract] OR "ambulatory care"[Title/Abstract] OR "mobile health unit*"[Title/Abstract] OR "community health clinic"[Title/Abstract] OR "community medicine"[Title/Abstract] OR "family medicine"[Title/Abstract] OR "family practi*"[Title/Abstract] OR "general practi*"[Title/Abstract] OR "ambulatory health cen*"[Title/Abstract] OR "primary medical care"[Title/Abstract] OR "community health cen*"[Title/Abstract] OR "family physician*"[Title/Abstract] OR "ambulatory care facilit*"[Title/Abstract] OR "ambulatory health facilit*"[Title/Abstract] OR "mobile health cen*"[Title/Abstract] OR "community health service*"[Title/Abstract] OR "public health"[Title/Abstract] OR "community health service*"[MeSH Terms] **AND** "malaysia"[MeSH Terms] OR "malaysia"[Title/Abstract] OR "perlis"[Title/Abstract] OR "kedah"[Title/Abstract] OR "penang"[Title/Abstract] OR "pulau pinang"[Title/Abstract] OR "perak"[Title/Abstract] OR "selangor"[Title/Abstract] OR "negeri sembilan"[Title/Abstract] OR "malacca"[Title/Abstract] OR "melaka"[Title/Abstract] OR "johor"[Title/Abstract] OR "johore"[Title/Abstract] OR "kelantan"[Title/Abstract] OR "terengganu"[Title/Abstract] OR "pahang"[Title/Abstract] OR "kuala lumpur"[Title/Abstract] OR "putrajaya"[Title/Abstract] OR "labuan"[Title/Abstract] OR "sabah"[Title/Abstract] OR "sarawak"[Title/Abstract] OR "klang valley"[Title/Abstract] | 414 |

1. **Scopus**

| **Terms** | **Results** |
| --- | --- |
| ( TITLE-ABS-KEY ( "malaysia*" OR "perlis" OR "kedah" OR "penang" OR "pulau pinang" OR "perak" OR "selangor" OR "negeri sembilan" OR "malacca" OR "melaka" OR "johor" OR "johore" OR "kelantan" OR "terengganu" OR "pahang" OR "kuala lumpur" OR "putrajaya" OR "labuan" OR "sabah" OR "sarawak" OR "klang valley" ) AND TITLE-ABS-KEY ( "diabet*" OR "hyperglyc*mia" OR "high blood glucose" OR "niddm" OR "noninsulin dependent diabetes mellitus" OR "non insulin dependent diabetes mellitus" OR "glucose metabolism disorders" OR "dysglyc*mia" OR "impaired glucose tolerance" OR "impaired fasting glucose" OR "maturity onset diabetes of the young" OR "mody" OR "t2dm" OR "type 2 diabetes" OR "type II diabetes" OR "maturity onset diabetes" OR "impaired fasting blood sugar" OR "hypertens*" OR "high blood pressure" OR "raised blood pressure" OR "hypercholesterol*" OR "hyperlipid*" OR "dyslipid*" ) AND TITLE-ABS-KEY ( "community health centers" OR "community medicine" OR "ambulatory care facilities" OR "ambulatory care" OR "mobile health units" OR "primary health care" OR "primary health care" OR "primary care" OR "public health" OR "ambulatory care" OR "mobile health unit*" OR "community health clinic" OR "community medicine" OR "family medicine" OR "family practi*" OR "general practi*" OR "ambulatory health cen*" OR "primary medical care" OR "community health cen*" OR "family physician*" OR "ambulatory care facilit*" OR "ambulatory health facilit*" OR "mobile health cen*" OR "community health service*" OR "public health" OR "community health service*" ) ) | 670 |

1. **EMBASE**

| **Terms** | **Results** |
| --- | --- |
| (('impaired fasting blood sugar':ti,ab OR 'maturity onset diabetes':ti,ab OR 't2dm':ti,ab OR 'mody':ti,ab OR 'maturity onset diabetes of the young':ti,ab OR 'impaired fasting glucose':ti,ab OR 'impaired glucose tolerance':ti,ab OR 'dysglycaemia':ti,ab OR 'dysglycemia':ti,ab OR 'disorders of glucose metabolism':ti,ab OR 'glucose metabolism disorder':ti,ab OR 'niddm':ti,ab OR 'high blood glucose':ti,ab OR 'hyperglycaemia':ti,ab OR 'hyperglycemia':ti,ab OR 'noninsulin dependent diabetes mellitus':ti,ab OR 'non insulin dependent diabetes mellitus':ti,ab OR 'type ii diabetes':ti,ab OR 'type 2 diabetes':ti,ab OR 'diabetes mellitus':ti,ab OR 'diabetes':ti,ab) OR ('hyperlipid*':ti,ab OR 'hypercholesterol*':ti,ab OR 'dyslipid*':ti,ab) OR ('hyperten*':ti,ab OR 'raised blood pressure':ti,ab OR 'high blood pressure':ti,ab OR 'hypertension':ti,ab)) AND ('klang valley':ti,ab OR 'sarawak':ti,ab OR 'sabah':ti,ab OR 'labuan':ti,ab OR 'putrajaya':ti,ab OR 'kuala lumpur':ti,ab OR 'pahang':ti,ab OR 'terengganu':ti,ab OR 'kelantan':ti,ab OR 'johore':ti,ab OR 'johor':ti,ab OR 'melaka':ti,ab OR 'malacca':ti,ab OR 'negeri sembilan':ti,ab OR 'selangor':ti,ab OR 'perak':ti,ab OR 'pulau pinang':ti,ab OR 'penang':ti,ab OR 'kedah':ti,ab OR 'perlis':ti,ab OR 'malaysia':ti,ab) AND ('community care':ti,ab OR 'community health service*':ti,ab OR 'ambulatory health facilit*':ti,ab OR 'mobile health cent*':ti,ab OR 'primary medical care':ti,ab OR 'ambulatory health cent*':ti,ab OR 'general pract*':ti,ab OR 'family pract*':ti,ab OR 'family medicine':ti,ab OR 'community health clinic':ti,ab OR 'public health':ti,ab OR 'primary care':ti,ab OR 'primary health care':ti,ab OR 'mobile health unit*':ti,ab OR 'ambulatory care':ti,ab OR 'outpatient department':ti,ab OR 'ambulatory care facilit*':ti,ab OR 'community medicine':ti,ab OR 'community health cent*':ti,ab OR 'primary healthcare':ti,ab) | 444 |

1. **MyMedR**

| **No.** | **Terms** | **Results** |
| --- | --- | --- |
|  | **Combined with “AND”** |  |
| 1 | "diabet*" OR "hyperglyc*mia" OR "high blood glucose" OR "niddm" OR "noninsulin dependent diabetes mellitus" OR "non insulin dependent diabetes mellitus" OR "glucose metabolism disorders" OR "dysglyc*mia" OR "impaired glucose tolerance" OR "impaired fasting glucose" OR "maturity onset diabetes of the young" OR "mody" OR "t2dm" OR "type 2 diabetes" OR "type II diabetes" OR "maturity onset diabetes" OR "impaired fasting blood sugar" OR "hypertens*" OR "high blood pressure" OR "raised blood pressure" OR "hypercholesterol*" OR "hyperlipid*" OR "dyslipid*" | 250 |
| 2 | "community health centers" OR "community medicine" OR "ambulatory care facilities" OR "ambulatory care" OR "mobile health units" OR "primary health care" OR "primary health care" OR "primary care" OR "public health" OR "ambulatory care" OR "mobile health unit*" OR "community health clinic" OR "community medicine" OR "family medicine" OR "family practi*" OR "general practi*" OR "ambulatory health cen*" OR "primary medical care" OR "community health cen*" OR "family physician*" OR "ambulatory care facilit*" OR "ambulatory health facilit*" OR "mobile health cen*" OR "community health service*" OR "public health" OR "community health service*" |  |
| 3 | "malaysia*" OR "perlis" OR "kedah" OR "penang" OR "pulau pinang" OR "perak" OR "selangor" OR "negeri sembilan" OR "malacca" OR "melaka" OR "johor" OR "johore" OR "kelantan" OR "terengganu" OR "pahang" OR "kuala lumpur" OR "putrajaya" OR "labuan" OR "sabah" OR "sarawak" OR "klang valley" |  |
