# Supplementary material for: Primary health care interventions targeting diabetes, hypertension or dyslipidemia in Malaysia: A scoping review
Source: PLoS One. 2026 Apr 20;21(4):e0346934. doi: 10.1371/journal.pone.0346934 (PMC13095119; doi:10.1371/journal.pone.0346934)
Supplement: S3 Table — (DOCX) [file pone.0346934.s003.docx]

## **S3 Characteristics of interventions* (n=32)**

| **Characteristics** | **Number of articles** | **Percentage** |
| --- | --- | --- |
| *Intervention Duration* |  |  |
| 1-2 days | 7 | 17.9 |
| 0-1 month | 1 | 2.6 |
| 1-6 months | 19 | 48.7 |
| 12-24 months | 5 | 12.8 |
| Not specified | 6 | 15.4 |
| *Intervention level* |  |  |
| Patients | 9 | 28.1 |
| Providers | 2 | 6.3 |
| Systems | 1 | 3.1 |
| Patients/Providers | 2 | 6.3 |
| Providers/Systems | 2 | 6.3 |
| Patients/Systems | 3 | 9.4 |
| Patients/Providers/Systems | 13 | 40.6 |
| *Chronic care model domains* |  |  |
| Delivery system design | 21 | 63.6 |
| Self-management support | 24 | 72.7 |
| Decision support | 8 | 24.2 |
| Clinical information system | 7 | 21.2 |
| Health system organization | 13 | 39.4 |
| Community linkages | 3 | 9.1 |
| Others/Unable to match | 1 | 3.0 |
| *Number of CCM domains targeted* |  |  |
| 1 | 10 | 31.3 |
| 2 | 6 | 18.8 |
| 3 | 8 | 25.0 |
| 4 | 5 | 15.6 |
| 5 | 2 | 6.3 |
| Not applicable | 1 | 3.0 |

** There were studies with the same intervention*
