# Supplementary material for: Primary health care interventions targeting diabetes, hypertension or dyslipidemia in Malaysia: A scoping review
Source: PLoS One. 2026 Apr 20;21(4):e0346934. doi: 10.1371/journal.pone.0346934 (PMC13095119; doi:10.1371/journal.pone.0346934)
Supplement: S4 Table — (DOCX) [file pone.0346934.s004.docx]

**S4 Descriptions of the interventions in terms of intervention levels and CCM domains**

| **First author (publication year)** | **Intervention name** | **Intervention period** | **Brief description of the intervention** | **Level of interventions** | | | **CCM domains** | | | | | | |
| --- | --- | --- | --- | --- | --- | --- | --- | --- | --- | --- | --- | --- | --- |
|  |  |  |  | **Patients** | **Providers** | **Systems** | **Delivery system design** | **Self management support** | **Decision support** | **Clinical information system** | **Health system organization** | **Community linkage** | **Others/ Unable to match** |
| Husin M (2023)(31) | EnPHC | 2 years (July 2017-June 2019) | EnPHC interventions include:  i) an Integrated Care Pathway;  ii) a patient visit checklist;  iii) Integrated Specialized Services by allied health professionals;  iv) NCD screening and cardiovascular risk stratification;  v) an NCD care form;  vi) the Family Health Teams concept;  vii) involvement of a care coordinator;  viii) pharmacist-led Cardiovascular Care Bundle Medication Therapy Adherence Clinic;  ix) clinical and prescribing audits;  x) structured communication across primary and secondary care levels with a fast track referral system | ⏺ | ⏺ | ⏺ | ⏺ | ⏺ | ⏺ | ⏺ | ⏺ | - | - |
| Lee JY (2020)(33) | Glucose telemonitoring | 6 months | - Participants are provided a gluco-telemeter and were instructed to transmit up to 6 glucose readings weekly, which will be uploaded to a central server.  - Participants received automated feedback on their glycemic and metabolic results.  - Monthly communications from the research team on self-management skills, blood glucose control, and the importance of medication adherence aimed at educating and motivating patients.  - Clinic visits at weeks 4, 12, and 24, where additional diabetes self-management education was given. | ⏺ | ⏺ | ⏺ | ⏺ | ⏺ | - | ⏺ | - | - | - |
| Ayadurai S (2018)(25) | Simpler tool | 6 months (27 weeks) | A multifaceted diabetes intervention tool (Simpler tool) - Statin or cholesterol control, insulin or glycemic control, medication, blood pressure, lifestyle, education, and cardiovascular risk prevention strategies.  The intervention group were given appointments to meet with the pharmacist trained with Simpler tool | ⏺ | ⏺ | ⏺ | ⏺ | - | - | - | - | - | - |
| Hasan UA (2019)(16) | Locally adapted and validated Diabetes Community Sharp Disposal Education Module | 3 months | The module consisted of four main topics; (1) medical sharps used for treatment of diabetes in the community; (2) proper handling of sharps prior to disposal; (3) improper community sharp disposal methods; (4) proper community sharp disposal method. The educational materials were in the form of lectures and demonstrations, flip charts and pamphlet, which were written in the local Malay language. | ⏺ | - | - | - | ⏺ | - | - | - | - | - |
| Muin MRA (2023)(15) | Prompt sheet for ED discussion and “Knowledge Translation Tools in the Management of Erectile Dysfunction” (LASTED) | Single session | The prompt sheet contained brief information on ED and options for ED discussion. The 4 options were: “I do not want to discuss ED;” “I want to discuss the risk of ED;” “I want to discuss treatment of ED;” and “I want to discuss the severity of ED.” The patients were asked to give the completed prompt sheet to their physician at the beginning of the consultation.  The consultation then proceeded with the physician using the LASTED flipchart based on the options selected on the prompt sheet. | ⏺ | ⏺ | ⏺ | ⏺ | ⏺ | ⏺ | - | - | - | - |
| Ismail M (2013)(19) | Self-monitoring blood glucose (SMBG) | 6 months | -Two-day classes that included practical demonstrations of SMBG and the usage of the glucometer was explained.  -Patients were supplied a glucometer with reagent test strips at no charge.  -Patients were advised to monitor their blood glucose levels, to keep a record in their logbooks, and adjust the dose of OHA/insulin accordingly.  -Required to visit their doctor at intervals of two months and to see the nurse every month to record their SMBG results. | ⏺ | - | ⏺ | ⏺ | ⏺ | - | - | ⏺ | - | - |
| Selvaraj FJ (2012)(39) | COACH program | 36 weeks | - COACH health booklet  - bi-weekly telephone follow-up by trained nurse educators for 24 weeks.  - phone calls and SMS to remind them about forthcoming follow-up visits | ⏺ | ⏺ | - | ⏺ | ⏺ | - | - | - | - | - |
| Norwati D (2023)(41) | Quran recitation audio | Single session | - Listen to the Quran recitation  - a copy of the Quran with Malay (native language) translation | ⏺ | - | - | - | - | - | - | - | - | ⏺ |
| Mohd Tahir NS (2023)(14) | Malay version Diabetes Conversion Maps (DCM) | Single session | - DCM topic was on insulin treatment initiation.  - The groups consist of 8-10 participants with one trained facilitator. Each session is about 60-90 minutes. The facilitator explored their baseline knowledge and their attitudes towards diabetes.The facilitator will read out loud the myth cards to stimulate a discussion. She then presented the facts cards in a clear, proper and concise way. | ⏺ | - | - | - | ⏺ | - | - | - | - | - |
| Selvadurai S (2021)(13) | Insulin injection technique re-education | 4 months | Patients were given intensive re-education during their monthly medication acquisition visits for 4 months. Each patient was given comprehensive education on their injection technique knowledge technique including LH physical examination and received an education kit consisting of a site rotation grid, educational insulin injection technique and LH leaflet during recruitment. | ⏺ | - | ⏺ | ⏺ | ⏺ | - | - | - | - | - |
| Tay CL (2021)(22) | Healthy Lifestyle Education (HLE) course | Single session | Healthy Lifestyle Education (HLE) is a structured group educational 3-hour course. The course is conducted by six different healthcare providers (doctor, pharmacist, medical assistant, nutritionist, occupational therapist and physiotherapist); with 30-minute allocation for each topic. | ⏺ | - | - | - | ⏺ | - | - | - | - | - |
| Nordin N (2020)(50) | Family Doctor Concept (FDC) | Not relevant (existing program) | The FDC offers personalized care. It is a restructuring of primary health services; its infrastructure and equipment, healthcare personnel, clinic’s floor set-up, clinic’s physical space and scheduled appointment; to ensure patients and population are taken care by specific Primary Healthcare Team (PHCT) according to “zone”. | - | - | ⏺ | ⏺ | - | - | - | ⏺ | - | - |
| Daud MH (2020)(28) | EMPOWER-PAR | 12 months | - Formation and training of the Chronic Disease Management team  - Intervention tools: Malaysian T2DM CPG, the QR, and the Global CV Risks Self-Management Booklet©  - Facilitation and support to implement the intervention | ⏺ | ⏺ | ⏺ | ⏺ | ⏺ | ⏺ | - | ⏺ | - | - |
| Heng WK (2019)(44) | Time of simvastatin | 4 months | -The defined time frames for after breakfast is between 6:00 am to 10:00 am; after dinner between 5:00 pm to 9.00 pm; and at bedtime accordingly. | ⏺ | - | - | - | ⏺ | - | - | - | - | - |
| Chai A (2020)(47) | Diabetes Medication Therapy Adherence Clinic (DMTAC) | 12 months | - Pharmacist incorporated the MTAC Diabetes Protocols in managing the medication and disease  - Patient was required to attend at least four DMTAC visits. | ⏺ | ⏺ | ⏺ | ⏺ | ⏺ | - | - | ⏺ | - | - |
| Ramli AS (2016)(29) | EMPOWER-PAR | 12 months | - Formation and training of the Chronic Disease Management (CDM) Team  - Distribution and utilisation of the intervention tools: Malaysian CPG and the Quick References (QR) on the Management of T2DM and the Global CV Risks Self-Management Booklet  - Facilitation and support to implement the intervention | ⏺ | ⏺ | ⏺ | ⏺ | ⏺ | ⏺ | - | ⏺ | - | - |
| Chee WSS (2017)(42) | Malaysian trans-cultural diabetes nutrition algorithm (tDNA) | 6 months | - Patients underwent an initial risk stratification checklist and were prescribed medical nutrition therapy (MNT) consisting of a structured low-calorie meal plan (1200 or 1500 kcal/day) and a physical activity prescription of at least 150 min per week.  - tDNA toolkit.  - The tDNA-MI subgroup received counselling that incorporated motivational interviewing principles.  - The tDNA-CC subgroup received conventional counselling techniques that focused on empathetic listening, education, persuasion, and encouragement. | ⏺ | ⏺ | - | ⏺ | ⏺ | - | ⏺ | - | - | - |
| Low WHH (2013)(36) | Community-Based Cardiovascular Risk Factors Intervention Strategies (CORFIS) | 6 months | - Allied health care team was constituted purposefully to support individual GPs.  - Each team member delivered care according to an agreed management and drug treatment protocol  - A custom designed, secure web-based application was set-up to capture patients’ data as well as to organise and coordinate care among all the healthcare providers.  - The trained allied health professionals counsel the patient monthly at their assigned GP clinics.  - Continuity of care for patients by the same care-provider was emphasised. - Home monitoring devices were loaned to the patients.  - Patients were provided with information on local patient associations and support groups. | ⏺ | ⏺ | ⏺ | ⏺ | ⏺ | ⏺ | ⏺ | - | ⏺ | - |
| Muhamad R (2012)(35) | Postprandial and fasting blood glucose monitoring | 6 months | All the subjects underwent the blood glucose monitoring protocols for the duration of 6 months with monthly follow up. | ⏺ | - | - | ⏺ | - | - | - | - | - | - |
| Wong SSL (2012)(30) | Colour-coded HbA1c-graphical record | 6 months | - Colour-coded HbA1c-graphical record which incorporates traffic light signal colours to denote HbA1c ranges.  - The HbA1c result was plotted on the graph at each follow-up (x-axis) and the trend was exhibited by drawing a line between the results. | ⏺ | - | - | - | ⏺ | - | - | - | - | - |
| Wong WJ (2020)(32) | Enhanced Primary Healthcare (EnPHC) | 17 months | EnPHC interventions include:  - Integrated specialised services (ISS)  - Cardiovascular care bundle medication therapy adherence counselling  - Clinical and prescribing audits  - Primary triage counter  - Secondary triage counter  - Care Coordinators  - Family Health Team (FHTs)  - NCD care form  - Identification of medication refill defaulter | ⏺ | ⏺ | ⏺ | ⏺ | ⏺ | ⏺ | ⏺ | ⏺ | - | - |
| Lim SC (2020)(23) | Steno REACH Certificate Course (SRCC) | 6 months | SRCC curriculum had ten modules and was designed to include independent online study and face-to-face classroom time | - | ⏺ | - | - | - | ⏺ | - | - | - | - |
| Lim PC (2023)(48) | Diabetes Medication Therapy Adherence Clinic (DMTAC) | Not relevant (existing program) | - medication initiation or discontinuations, dosage adjustments, and recommendations for laboratory investigations.  - pharmacists were granted authorization by the physicians to carry out insulin dosage adjustments  - DMTAC pharmacists routinely documented patients’ medical, social, and family histories, drug-related issues, interventions, laboratory data, treatment regimens, and adherence using the Malaysian Medication Adherence Tool (MyMAAT) score | ⏺ | ⏺ | ⏺ | ⏺ | ⏺ | - | - | ⏺ | - | - |
| Tay CL (2022)(18) | Multi-faceted intervention to increase ED screening | One meeting & one-day workshop | - an audit and feedback and a mandate from management  - 1-day workshop on men’s sexual health included short lectures on ED screening, assessment and management, case scenarios, role play, group discussions and presentations, and interactive question-and-answer sessions before and after the workshop. | - | ⏺ | ⏺ | ⏺ | - | ⏺ | - | ⏺ | - | - |
| Ayadurai S (2020)(24) | Simpler Tool | 6 months (27 weeks) | A multifaceted diabetes intervention tool (Simpler tool) - Statin or cholesterol control, insulin or glycemic control, medication, blood pressure, lifestyle, education, and cardiovascular risk prevention strategies.  The intervention group were given appointments to meet with the pharmacist trained with Simpler tool | ⏺ | ⏺ | ⏺ | ⏺ | - | - | - | - | - | - |
| Sazlina SG (2015)(40) | Personalized feedback (PF) with or without peer support (PS) | 3 months | - PF received feedback comprised participants’ physical activity patterns.  - PS group received support from peer mentors | ⏺ | ⏺ | ⏺ | ⏺ | ⏺ | - | - | ⏺ | - | - |
| Lee JY (2015)(34) | Diabetes telemonitoring | 7 weeks | TG participants received a web-enabled glucometer that enables self-monitoring and goal setting. Recorded readings were transmitted to an online portal. Participants received feedback upon receipt of transmission when there were three continuous blood glucose values of ≤3.9 mmol/l and ≥11.1 mmol/l. | ⏺ | - | - |  |  |  |  |  |  |  |
| Ahmad S (2016)(12) | Insulin injection technique education | 20 minutes | - Education on proper insulin injection technique was delivered using placebo injection device.  - Individualization of education | ⏺ | - | - | - | ⏺ | - | - | - | - | - |
| Bondi ME (2020)(17) | Educational intervention module (wound) | 2 days | The educational intervention module was developed, and adapted from the Wound Care Guidelines consisted of three main sections:   - Basic wound principle - Concept of wound care management - Principle aspect of wound care management. | - | ⏺ | - | - | - | ⏺ | - | - | - | - |
| Lee XY (2015)(49) | Diabetes Medication Therapy Adherence Clinic (DMTAC) | NA (4 sessions) | - pharmacists collaborate with physicians providing the services.  - Patients with poor glycaemic control undergo follow up counselling sessions for a minimum of four visits. | ⏺ | ⏺ | ⏺ | ⏺ | ⏺ | - | - | ⏺ | - | - |
| Chew BH (2018)(27) | Value-based emotion-focused educational program (VEMOFIT) | 6 weeks (booster session at week 18) | - Nurses from the participating health clinics are invited to participate in a 2-days training course.  - Each participant in the VEMOFIT program was allowed to bring along one significant other person as a co-participant.  - The VEMOFIT program included:  (1) exploring personal beliefs regarding diabetes;  (2) training emotional skills; and  (3) providing social support and setting short- and long-term goals. | ⏺ | ⏺ | ⏺ | ⏺ | ⏺ | - | - | ⏺ | ⏺ | - |
| Chew BH (2019)(26) |  |  |  |  |  |  |  |  |  |  |  |  |  |
| Chow EP (2015)(20) | Pharmacist-led home-based diabetes education | 3 months | - Home visits by a trained pharmacist with home-based educational intervention (proper use of medications and on T2DM)  - Patients were given an information booklet on T2DM and a food pyramid chart prepared by the Ministry of Health, Malaysia.  - Medication charts and pictorial labels for each of the medications were given when necessary.  - Telephone reminders for monthly prescription refills and reassessments of medication use. | ⏺ | ⏺ | ⏺ | ⏺ | ⏺ | - | ⏺ | ⏺ | - | - |
| Nordin N (2021)(46) | Family Doctor Concept (FDC) | Not relevant (existing program) | - The FDC restructures the primary healthcare services, whereby patients and population are taken care of by a specific primary healthcare team (PHCT) according to zone.  - “One Family One Doctor” concept | - | - | ⏺ | ⏺ | - | - | - | ⏺ | - | - |
| Ngah NF (2020)(43) | Retinal Disease Awareness Program (RDAP) | Not relevant (existing program)) | - Retinal Disease Awareness Program (RDAP) was initiated to enhance the existing screening programs conducted by the local health clinics.  - The eye examination was conducted by the ophthalmology team with visual acuity testing and fundus photography for diabetic retinopathy (DR) assessment.  - A trained photographer took retinal images and sent them to a remote trained reader (typically an ophthalmologist or optometrist) for interpretation. | ⏺ | ⏺ | ⏺ | ⏺ | - | - | - | ⏺ | - | - |
| Chua SS (2012)(37) | Pharmacist counselling (a component of Community-Based Cardiovascular Risk Factors Intervention Strategies (CORFIS)) | 6 months | - The study involved a group of pharmacists, dietitians and nurses. Each participant was interviewed and counselled by one of the pharmacists, dietitians and nurses at the GP clinic.  - Each participant spent about 30 to 60 min with each of the healthcare providers.  - During the 24-week follow-up assessments, the pharmacist reviewed the participants’ medications and counselled the participants every 4 weeks, noted any PCIs encountered by the participants and helped to resolve the PCIs.  - If required, the pharmacist would contact the GP concerned to alert, discuss and if possible, to resolve the PCIs which could affect the participants’ clinical outcomes.  - The dietitians provided dietary advice while the nurses advised the patients on general healthcare such as foot care. | - | ⏺ | ⏺ | ⏺ | ⏺ | - | - | - | - | - |
| Tan LK (2023)(45) | Malaysia Healthy Plate (MHP) | Not relevant (existing program) | -MHP was introduced by the MOH Malaysia in 2016 with the Tagline "Suku suku separuh" (Quarter Quarter Half)  - A healthy plate is comprised of a quarter plate of grains, followed by a quarter plate of and a half plate of fruit and vegetables | ⏺ | - | ⏺ | - | ⏺ | ⏺ | - | - | ⏺ | - |
| Tajudin TR (2020)(38) | Diabetes Awareness Day & diabetes clinic (primary care-based behaviour modification program) | 6 months | - Diabetes awareness day: participants were offered free blood tests, meals prepared by a caterer with a strong background of diabetes food preparation and there were several talks on diabetes and its management.  - Diabetes clinic held once a week with a patient load of five to seven patients per session.  - Patients were followed up every month for diabetic treatment and management for a total of six months. | ⏺ | ⏺ | ⏺ | ⏺ | ⏺ | - | - | ⏺ | ⏺ | - |
| Lee SS (2022)(21) | DePEC (Dementia Prevention and Enhanced Care)-Nutrition trial | 8 months | Complex dietary and behavioural intervention evaluating the feasibility of the combination of salt intake reduction and increased high-nitrate vegetable consumption among middle-aged and older Malaysian adults with elevated blood pressure  1. Group nutritional counselling  2. Information booklets  3. A salt measuring spoon (salt intervention)  4. Biweekly text messages.  5. Reinforcement video messages | ⏺ | ⏺ | ⏺ | ⏺ | ⏺ | - | ⏺ | ⏺ | - | - |
