# Supplementary material for: Primary health care interventions targeting diabetes, hypertension or dyslipidemia in Malaysia: A scoping review
Source: PLoS One. 2026 Apr 20;21(4):e0346934. doi: 10.1371/journal.pone.0346934 (PMC13095119; doi:10.1371/journal.pone.0346934)
Supplement: Attachment — Submitted filename: Response to reviewer.docx [file pone.0346934.s006.docx]

| **Reviewer comments** | **Feedback from authors** |
| --- | --- |
| Reviewer #2: The authours have attepted a topic of importance and they need to be coended for that. But having said that, it is presented in a very confusing and shabby manner. THe table sumarising the studies and the interventions are too large and confusing.  THE main objective namely, the relationship of the interventions individually to the coponents of the primary care model is not presented cllearly. It is hard to read and understand what they are trying to say | Thank you for your comment. We have revised Table 2 to improve its clarity and presentation. Specifically, we have reduced the number of columns to make it more concise and easier to read. In addition, we have edited the Results column to enhance readability and ensure the findings are presented more clearly.  One of the objectives of the study is to summarise the intervention components, where it is now shown in Table 2, together with the description of the interventions. |
